# Extraventricular Neurocytoma of the Posterior Fossa: A Case Report Written by ChatGPT

**DOI:** 10.7759/cureus.35850

**Published:** 2023-03-06

**Authors:** Ajay Hegde, Siddharth Srinivasan, Girish Menon

**Affiliations:** 1 Neurosurgery, Kasturba Medical College, Manipal, IND

**Keywords:** chatgpt, brain tumour, neuro oncology, artificial intelligence (ai), extraventricular neurocytoma

## Abstract

ChatGPT (Chat Generative Pre-trained Transformer) is the latest artificial intelligence (AI) tool that has taken the internet by storm. A large language model-based AI tool, ChatGPT is used to generate natural prose-like answers learning from a large database of text. A lot is being written and spoken about the future of academic writing using this tool.We attempt to generate a short case report of a brain tumour labelled extraventriuclar neurocytoma (EVN) which is a rare tumour of the central nervous system. When posed with a set of questions on this uncommon topic, ChatGPT did well with relevant but not very specific answers. Validation of the information is an issue like with most machine learning solutions. This case report is an attempt to summarise EVN and its management.

Q: Summarise extraventricular neurocytoma.

ChatGPT:* EVN is a rare and benign tumor that typically occurs in the brain. It often causes no symptoms, but as it grows, it can cause headaches, seizures, and changes in vision or balance. Surgery is usually curative, but there is a lack of studies to know the best treatment options. The prognosis is generally good with high rates of long-term survival and low rates of recurrence.*

Text *italicised* in the article is generated by ChatGPT.

## Introduction

*Extraventricular neurocytoma (EVN) is a rare, benign tumor of the nervous system that primarily arises from the cells of the neuroepithelium. EVN is a slow-growing tumor that often causes no symptoms for a long time, but as it grows, it can cause symptoms such as headaches, seizures, and changes in vision or balance. The diagnosis of EVN is often made by imaging studies such as MRI or CT scans. While most EVNs occur in the supraentorial compartment of the brain, a small subset of these tumors can occur in the posterior fossa. Posterior fossa EVNs are considered to be an extremely rare subtype of this tumor, and as such, there is limited information available on their epidemiology, clinical presentation, and management.* *In this report, we aim to provide an overview of the current knowledge on EVN, including epidemiology, clinical presentation, diagnosis, management, and prognosis using *ChatGTP (Chat Generative Pre-trained Transformer) which is a popular natural language processing (NLP) based machine learning model which is are able to understand, interpret, and generate human language.

## Case presentation

**Figure 1:**
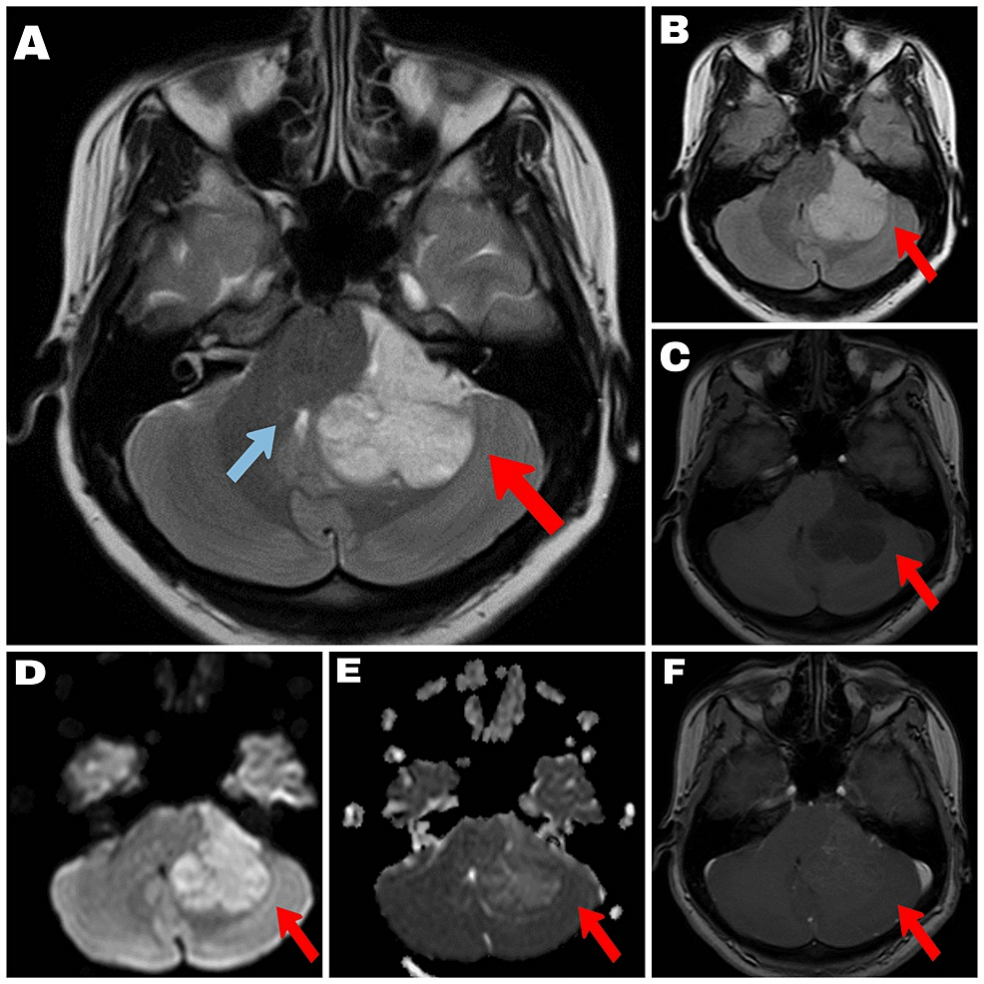
MRI characteristics of the lesion A 4cm x 4.5cm extra-axial space occupying lesion in the left cerebellopontine angle cistern, compressing the left cerebellar hemisphere , medulla, pons and mid brain. The lesion is not causing internal auditory meatus widening/extension, no white matter edema and has the following MRI characteristics: A- T2: Homogenous hyperintense; B- FLAIR: Homogenous hyperintense; C- T1: Homogenous hypointense; D- DWI: Homogenous hyperintense; E- ADC: Homogenous isointense; F- T1 contrast: Minimal heterogenous enhancement with few cystic areas. Red arrow demonstrates the lesion in all sequences, blue arrow in sequence A depicts brain stem distortion with compression of the fourth ventricle.

A 25-year-old lady presented with complaints of headache for a duration of one year, aggravated for the last few weeks associated with nausea and occasional vomiting. She had a recent onset gait imbalance and left sided tinnitus for two weeks which triggered further evaluation. On examination her cranial nerves were intact but for a gaze-evoked fast beating nystagmus to the left on ocular examination (Bruns nystagmus). Gait instability was present with positive cerebellar signs on the left. Computed tomography (CT) of the brain showed an isodose lesion in the left cerebellopontine angle which prompted further evaluation with magnetic resonance imaging (MRI). On MRI scans the lesion was a broad-based lesion occupying the left cerebellopontine angle with extension into the foramen of Lushka. It was hypointense on T1, hyperintense on T2 and T2 flair and showed minimal enhancement on T1 post contrast images. Diffusion-weighted imaging showed high signal (Figure 1). The differential diagnoses considered were ependymoma, primitive neuroectodermal tumours or a medulloblastoma.

**Figure 2:**
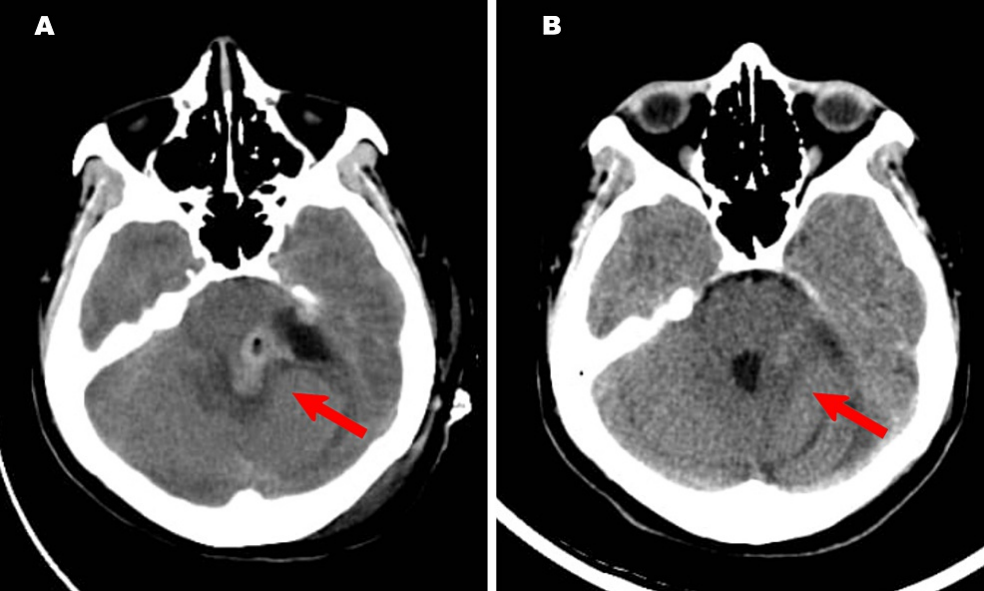
Postoperative CT scan images A- Immediate postoperative non-contrast axial CT scan showing tumour decompression; B- Three-month follow-up non-contrast CT scan showing good clearance of the lesion. Red arrow identifies the operative field.

The patient was consented and planned for surgery. A left retromastoid craniectomy and tumour debulking was done. The lesion was found to be extremely vascular and had poorly defined planes along the medial surface to the cerebellum and brain stem. The cranial nerves were within the lesion and safe debulking was done under monitoring of the seventh cranial nerve. Postoperatively the scan showed near total tumour decompression and patient was discharged on the seventh postoperative day with no new neurological deficits (Figure 2). She returned to work in six weeks. Histopathology findings were of a tumour composed of ovoid to cuboidal cells arranged in a peritheliomatous pattern with uniform round vesicular nuclei and < 1 mitosis/high power field. Immunohistochemistry was positive for synaptophysin and neuronal nuclear antigen (NeuN), patchy for S100, focal for transducin-like enhancer (TLE) and negative for glial fibrillary acidic protein (GFAP), epithelial membrane antigen (EMA), progesterone receptor (PR), desmin, CD99, GATA 3, signal transducer and activator of transcription 6 (STAT6) and cytokeratin (CK). Ki67 index was 4% at hotspots with a final diagnosis of extraventricular neurocytoma. 

## Discussion

Neurocytoma is a rare type of brain tumor that originates from the cells that form the supportive tissue of the nervous system, known as neuroglial cells. It can occur in both the central nervous system (CNS) and the peripheral nervous system (PNS). Neurocytomas are usually benign, but can have aggressive biological behaviour in some cases. There are two main subtypes of neurocytomas: intraventricular neurocytoma (IVN) and extraventricular neurocytoma (EVN). IVN is a type of neurocytoma that occurs within the ventricles of the brain, while EVN is a type of neurocytoma that occurs outside the ventricles. The exact incidence of EVN is not well defined, but it is estimated to account for approximately 5-10% of all neurocytomas. The annual incidence of neurocytomas is estimated to be less than 2 cases per million people. 

*Despite sharing many similarities, there are some important differences between CN and EVN in terms of presentation, age, and prognosis. CNs are typically diagnosed in young adults in their third or fourth decade of life, with symptoms such as headache, nausea, vomiting, and changes in cognitive function. EVNs are diagnosed at a slightly earlier age, often in the second decade of life and may present with symptoms such as seizures or changes in motor function. CNs have a more favourable prognosis compared to EVNs, with a 5-year survival rate of approximately 95%, while EVNs have a 5-year survival rate of approximately 80%. *The pathological differences between the two are summarised in Table 1.

**Table 1:** Histopathological and molecular differences between central neurocytoma (CN) and extra ventricular neurocytoma (EVN)

Feature	Central Neurocytoma (CN)	Extraventricular Neurocytoma (EVN)
Histopathology	Round or oval cells with eosinophilic cytoplasm and well-defined cell borders	More variable appearance, with cells ranging from round to spindled and variable cytoplasmic appearance
Molecular Profile	Mutations in the beta-catenin gene (CTNNB1)	Mutations in the MYC gene
Chromosomal Alterations	Gains of chromosomes 7 and 17, losses of chromosomes 1, 2, and 13	Gains of chromosomes 1, 7, and 17, losses of chromosomes 13 and 19
Immunohistochemistry	Strong immunoreactivity for neural markers (e.g., neurofilament, synaptophysin)	More variable staining patterns for neural markers

*The potential differential diagnoses for a posterior fossa lesion as in our case are medulloblastoma, cerebellar astrocytoma, ependymoma, meningioma, brainstem glioma, hemangioblastoma and posterior fossa extraventricular neurocytoma (EVN). Symptoms of posterior fossa EVN can vary depending on the location and size of the tumor, but may include headaches, ataxia, cranial nerve deficits, and other neurological symptoms. **Diagnosis of posterior fossa EVN is typically made by imaging studies such as MRI, which can show the characteristic features of the tumor.*** EVN is most often a postoperative histopathology diagnosis. On preoperative MRI images, it is usually a differential. The diagnosis of brain tumours is done on MRI images and perhaps ChatGPT provided that answer without going into the specifics. 

The characteristic imaging features of EVN on MRI, On T1-weighted images, EVNs typically have low signal intensity, while on T2-weighted images, they have high signal intensity. After contrast administration, most EVNs show intense and homogeneous enhancement. They usually have a clear boundary with the surrounding brain tissue, and do not invade adjacent structures. It is important to note that these imaging features are not specific to EVN, and similar findings can be seen in other brain tumors, making a definitive diagnosis often requires a combination of imaging findings, clinical presentation and pathological examination. Amongst the other differentials, Medulloblastomas typically have a higher signal intensity on T1-weighted images and a lower signal intensity on T2-weighted images, consistent with a solid lesion. Ependymomas are often seen as cystic lesions on MRI with variable signal intensity, but they typically have a characteristic peritumoral rim enhancement on contrast-enhanced images. 

*Surgical resection is the primary treatment for posterior fossa EVN, and complete resection is often possible. However, the posterior fossa is a complex area of the brain, and surgery in this region can be challenging and associated with a higher risk of complications. Prognosis for posterior fossa EVN is generally good, with high rates of long-term survival and low rates of recurrence. However, due to the rarity of this disease, there is a lack of consensus on the optimal management and treatment options for patients with posterior fossa EVN. Adjuvant treatments for extraventricular neurocytoma (EVN) can include a combination of surgical resection, radiation therapy, and chemotherapy. The goal of adjuvant treatment is to reduce the risk of recurrence of the tumor after initial surgical resection. The choice of adjuvant treatment will depend on a variety of factors, including the size and location of the tumor, the extent of surgical resection, and the patient's overall health. Chemotherapy may also be used in conjunction with radiation therapy for the treatment of EVN. Chemotherapy drugs for EVN may include temozolomide, carboplatin, or other agents. A multidisciplinary team of doctors, including a neurosurgeon, radiation oncologist, and medical oncologist, should work together to determine the best course of treatment for each individual patient. **One study published in the Journal of Neurosurgery found a recurrence rate of 2.5% for EVNs that were completely resected. Another study published in the Journal of Clinical Neurosciences found a recurrence rate of 4.5% for completely resected EVNs, and a recurrence rate of 27% for subtotally resected tumors. A study published in the Journal of Neuro-Oncology found a recurrence rate of 3.2% for EVNs that were completely resected, and a recurrence rate of 17.6% for subtotally resected tumors.*** The above facts could not be verified and recurrence rates varied from 4% to 50% in literature with atypical variants and subtotal resections having higher rates of recurrence [1-3]. ChatGPT was not incorrect but rather could not be verified. 

Differentiating typical and atypical extraventricular neurocytomas (EVNs) can be challenging, as there is often overlap between the two subtypes in terms of their clinical, radiologic, and histologic features. However, there are several key differences that can be used to differentiate between typical and atypical EVNs. One of the main differences between typical and atypical EVNs is the degree of malignancy. Atypical EVNs tend to grow more rapidly and are more likely to recur after surgical resection than typical EVNs. Another key difference between typical and atypical EVNs is their histologic appearance. Typical EVNs are composed of small, round cells that form small clusters or rosettes. Atypical EVNs, on the other hand, tend to have a more complex histologic appearance, with cells that are larger and more abnormal in shape and arrangement. Atypical EVNs may also have a higher degree of nuclear atypia and increased mitotic activity, which are markers of increased malignancy. MRI imaging can also be helpful in differentiating between typical and atypical EVNs. Typical EVNs are typically well-circumscribed and have a cystic appearance on MRI, while atypical EVNs tend to be more irregular in shape and have a more solid or partially cystic appearance. In general, the treatment approach for typical and atypical EVNs is similar, with surgical resection being the primary treatment for both subtypes. However, the use of adjuvant therapy, such as radiation therapy or chemotherapy, may be more commonly recommended for atypical EVNs due to their higher degree of malignancy.

ChatGPT is a large language model (LLM) machine learning system that autonomously learns from data and can produce sophisticated and seemingly intelligent writing after training on a massive data set of text [4]. The information we extracted was based on specific questions (Table 2) and considering the rarity of the disease extraventricular neurocytoma, ChatGTP did a reasonable job of producing relevant output in most circumstances. The text produced was largely unplagarised with a similarity of 10% using the popular platform Turnitin^TM^. The matches were commonly used medical terms rather than sentences and were indexed from medical literature. 

**Table 2:** Questions posed to ChatGPT to generate this manuscript.

Questions posed to ChatGPT
Introductory paragraph for a journal article on extraventricular neurocytoma
Posterior fossa extraventricular neurocytoma
MRI imaging features of extraventriuclar neurocytoma
What would be the differential diagnosis for a 25 year old lady, presenting with headache and vomiting and MRI showing a posterior fossa lesion in the brain
Incidence of extra ventricular neurocytoma
Create a table on the histopathological and molecular differences between central neurocytoma and extra ventricular neurocytoma
How to differentiate extraventricular neurocytoma from medulloblastoma and ependymoma on MRI imaging?
What are the adjuvant treatments available for extraventricular neurocytoma?

**Figure 3:**
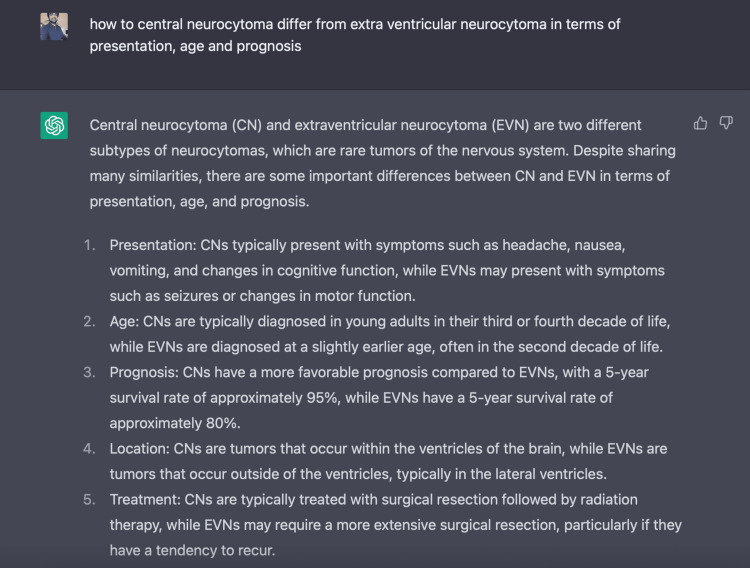
The use of ChatGPT in manuscript preparation

**Figure 4:**
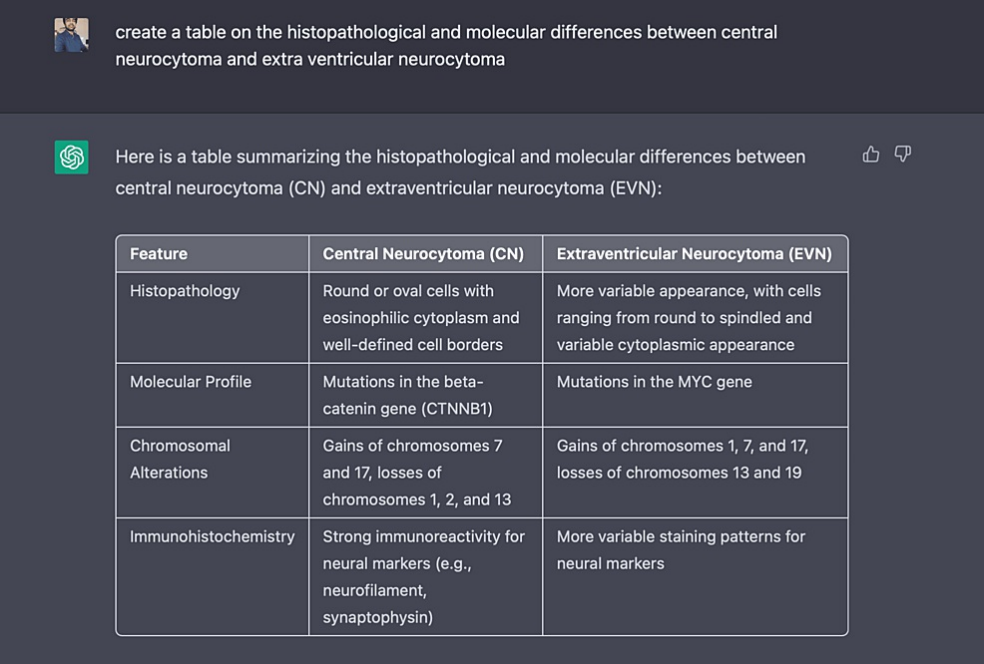
Creating a table with ChatGPT

Figure 3 and Figure 4 demonstrate the use of the ChatGPT platform.

However, when asked specific questions requiring a number or a value the output could not be verified due to the lack of referencing. This could degrade the quality and transparency of research and fundamentally alter our autonomy as human researchers [5]. Machine learning systems are a black box with little information of what goes through the process of generating the output. ChatGPT is no different as the results are fluent reproductions of selected works with no clear methodology of selection or citing the referring source, which is a drawback in writing scientific articles. It could be used to write introductory paragraphs or specific sections of a scientific article that requires fewer referencing, but lacks credibility when presented with questions that require an in-depth understanding of the literature. In such circumstances ChatGPT often generated simple answers with constant repetition of known facts. Such errors could be due to an absence of the relevant articles in ChatGPT’s training set, a failure to distill the relevant information or being unable to distinguish between credible and less-credible sources. It seems that the same biases that often lead researchers astray, such as selection, availability and confirmation biases, are reproduced and often even amplified in conversational artificial intelligence [6].

## Conclusions

*In summary ChatGPT concluded that, Posterior fossa extraventricular neurocytoma is an extremely rare subtype of EVN, which affects the base of the brain. The clinical presentation, diagnosis, and management of these tumors is similar to that of EVN located elsewhere in the brain, but the surgery is more complex and challenging. The prognosis is generally good, but more studies are needed to understand this rare subtype of EVN. *ChatGPT can be used with supervision to stitch together a well-written manuscript. However, authors should be cautious as it can easily mislead them with well-written text and distorted facts.
